# Development and validation of a self-report questionnaire to assess executive functions in healthy adults: the executive functions in healthy adults questionnaire (EFHAQ)

**DOI:** 10.1038/s41598-026-65768-y

**Published:** 2026-08-08

**Authors:** Ines Pfeffer, Tilo Strobach, Christina Niermann

**Affiliations:** 1 Department of Pedagogy, Medical School Hamburg, Hamburg, Germany; 2 Institute of Cognitive and Affective Neuroscience (ICAN), Medical School Hamburg, Hamburg, Germany; 3 Department of Psychology, Medical School Hamburg, Hamburg, Germany; 4 Institute of Physical Education and Sport Science, Osnabrück University, Osnabrück, Germany

**Keywords:** Executive functions, Self-regulation, Self-report questionnaire, Inhibition, Updating, Shifting, Health care, Psychology, Psychology

## Abstract

Aim of this research was to develop and validate a time-efficient, ecologically valid self-report questionnaire measuring executive functions in healthy adults, grounded in the unity/diversity framework of Miyake (i.e., inhibition, updating, shifting). Across a pre-study and three main studies (*N*_*total*_ = 1.574), an initial 55-item pool was refined to a final 15-item set. Exploratory and confirmatory factor analyses (Studies 1–3) revealed, while the data did not support the expected three-factor model, a stable five-factor structure with the factors flexibility, focusing, inhibition, adaptation, and weighing up. The subscales demonstrated acceptable internal consistencies (Studies 1–3) and test-retest reliability over three weeks (Study 3). Construct validity was supported by significant correlations with established measures of self-regulation abilities (Studies 1–3). Criterion validity was shown by associations with life satisfaction and affective well-being (Study 3). Although limited by a predominantly young, highly educated sample, the questionnaire provides a reliable and valid open-access tool for examining the role of executive functions in health behavior, academic achievement or social functioning.

**Supplementary Information:**

The online version contains supplementary material available at 10.1038/s41598-026-65768-y.

## Introduction

Self-regulation is an important factor of human functioning, which supports the modification of thoughts, feelings, impulses, and behaviors in the service of long-term goals^1^. Self-regulation is relevant for goal striving in various contexts such as health behavior change (e.g. exercise behavior change, healthy diet, alcohol consumption, smoking cessation), academic achievement or social functioning. From a neuropsychological point of view, self-regulation seems to highly depend on the frontal lobes of the brain^2^; these brain regions support mechanisms of self-regulatory goal pursuit such as active representation of a self-regulatory goal, active inhibition of impulses or flexibly switching between different means and goals^3^. Abilities subserved by the frontal lobe are often labeled as executive functions (EFs). However, an important prerequisite for studies examining associations between EFs and self-regulation of health behaviors are reliable and valid measurement tools. In the present paper, our aim is to develop and validate such a tool to measure EFs in a self-report way in healthy adults.

## Executive functions

EFs refer to cognitive processes that enable top-down control of behavior and adaptive functioning such as organizing, directing, and managing cognitive activities or emotional responses. These multifaceted functions involve higher-order control of lower-order (sub-)processes. Using a structural equation modeling approach, Miyake and colleagues^4,5^ integrated the opponent theoretical views of a unitary EF system^6–9^ and a modular view assuming a set of several distinct EFs^10,11^. The authors found evidence supporting both the unity and diversity of executive control (i.e., the prominent unitary/diversity framework), that categorizes EFs into three processing domains: inhibition, updating, and shifting. *Inhibition* involves overriding dominant responses, *updating* relates to monitoring and manipulating working memory contents, and *shifting* allows flexible switching between different tasks and activities. With regard to goal striving and self-regulation of human behavior, successful self-regulation relies, for example, on inhibiting behaviors, such as incompatible or inappropriate habits or impulses. Updating helps to maintain mental representations of self-regulatory goals and means to achieve them. High shifting ability allows individuals to abandon suboptimal plans and pursue alternative means (means-shifting). Additionally, goal-shifting permits temporary disengagement from self-regulatory goals to explore tempting alternatives^3^.

### Measuring executive functions

Performance-based measures evaluate EFs through structured tasks, focusing on performance-based efficiency. They are highly standardized and occur in well-controlled environments. Various experimental computer tasks serve as indicators of the three fundamental EFs (for a meta-analysis^12^. Inhibition is frequently assessed by the Stroop task, the Go-NoGo task, the Simon task, or the Flanker task^13–17^. *N*-back measures^18,19^ have been found to reflect primarily the updating function. Shifting is operationalized with task switching paradigms^20–22^. These performance-based tasks provide increased objectivity and help researchers to understand individual EF differences in the lab.

However, with regard to the impact of EFs on daily functioning, several researchers^23,24^ raised an important point regarding the limitations of performance-based tasks in assessing EFs. That is, these tasks often break down an integrated system into artificial components, where the examiner has set the goals and outcomes for the testing session. Thus, these tasks are missing the multidimensional, context-dependent nature of real-world decision-making^25–27^. Therefore, these tasks show limited ecological validity, i.e., the extent to which findings apply to everyday situations. Further limitations of these measures are that they might assess state-like performance at a given point in time (as opposed to trait-like EFs^28^ as well as they are time-consuming and resource-intensive (i.e., they require specialized computer equipment and trained professionals). Therefore, developing measures that are less resource-intensive and better capture trait-like EFs related to real-world environments seems to be crucial. One potential way to achieve this goal is to apply self-report rating scales.

### Measuring executive functions with self-report rating scales

Subjective measures of EFs rely on an individual’s evaluation of behavior across different contexts and over a longer period^29^. These measures were designed to offer a practical way to assess EFs in real-world problem-solving situations and they are closely linked to the processes evaluated by performance-based EF tasks^24^. In general, Corneille and Gawronski^30^ argue that self-report measures compared to performance-based tasks, are often the better option to measure psychological constructs, such as thoughts, feelings, and cognitive processes. The authors assume that self-report measures have fewer disadvantages than generally assumed in terms of contamination by external influences (e.g., social desirability), the accessibility to unconscious content (e.g., access to unconscious thoughts and feelings), the assessment of automatic processes (e.g., suitable to measure automatic processes) or robustness (e.g., stability over time at the individual level due to new information). Furthermore, the authors highlight several advantages of self-reports such as higher reliability, better predictions of behavior and more flexibility in capturing lived experiences and therefore recommend a sophisticated use of self-reports and caution in using performance-based measures in basic and applied research.

In the field of EFs, several of those subjective measures were developed. Importantly, however, these measures were mainly generated for clinical populations (e.g., ADHD, brain injury; for an overview see^24^. Examples of widely used instruments are the BRIEF-A (Behavior Rating Inventory of Executive Function – Adults Version^31^, the BADS (Behavioural Assessment of the Dysexecutive Syndrome^32^, the ADEXI (Adult Executive Functioning Inventory^33^, as well as the DEX and DEX-R (Dysexecutive Questionnaire and Dysexecutive Questionnaire-Revised^23,34^. To assess EFs, these existing measures, however, often contain items that reflect pathological behaviors, which do hardly apply to healthy individuals (e.g., difficulties in realizing the extent of my health problems; talking about events that never happened^29^. Therefore, the validity of these existing measures for healthy adult populations is questionable, although EFs are also relevant for the self-regulation of everyday behavior in these populations. Furthermore, the existing EF self-report measures were not constructed based on subscales (e.g., the empathy subscale of the Executive Function Index [EFI]^35,29^ that map the widely accepted and used theoretical EF taxonomies, such as the one of Miyake and colleagues^4,5^.

EFs, assessed via performance-based as well as self-report questionnaire measures, are barely correlated with each other^24^. In addition, both measures are associated with different aspects of functioning in everyday life such as social functioning^36^, and behavioral problems^37,38^, indicating that these two types of measures should not be considered equivalent or interchangeable. Self-report rating scales and performance-based measures of EFs serve different purposes and provide distinct insights and should therefore be applied complementary. Thus, an approach that combines both types of assessment may provide a more comprehensive understanding of EFs in everyday behavior^39,40^. However, there is currently no questionnaire based on Miyake’s EF concept specifically for healthy adults. Therefore, developing such an ecologically valid self-report assessment that maps onto parallel performance-based models and measures of EFs is necessary to advance our understanding of health behaviors, academic / professional success or social functioning. Developing an ecologically valid self-report assessment for healthy adults that maps onto could be valuable for further research in health behavior, academic, and professional success or social functioning. As EFs might contribute to self-regulatory outcomes in different ways (e.g., as a predictor, moderator, or mediator^3^ a series of exemplary research questions can be examined: (1) Are EFs associated with emotion regulation strategies? (2) Are people with higher EFs better at translating intentions into action? (3) Does training of EFs lead to better executive functioning and does this improvement transfer to self-regulation mechanisms in everyday life?

### The present research

EFs seem to play a critical role in the self-regulation of, e.g., health behavior change, translating behavioral intentions into action and pursuing long-term goals^3^. Further, self-report questionnaires are useful for screening and gathering subjective information about EFs. However, there is currently no such questionnaire for healthy adults based on a theoretically sound EF model, such as the model of Miyake and colleagues^5,4^. The model of Miyake and colleagues was chosen because it is practical and widely used for understanding EF at the behavioral level (compared to models at the neural level requiring neuroimaging measures), and focuses on commonalities as well as diversity in EF by differentiating common and specific components (unity/diversity)^39^. We aim to provide a self-report instrument that maps as best as possible onto the model of Miyake to enable the complementary examination of everyday behaviors based on the combination of performance-based tasks and self-report questionnaires. Therefore, the aim of our study was to develop and validate an ecologically valid self-report questionnaire for healthy adults assessing EFs in the context of this model: the Executive Functions in Healthy Adults Questionnaire (EFHAQ). We also aim to provide a time efficient open access tool for healthy adults, which is applicable for research across various contexts such as health behaviors and health behavior change (e.g., exercise behavior, healthy diet, alcohol consumption, smoking cessation) academic achievement or social functioning. To do so, in the remainder of this paper, we present a pre-study including item generation and scale development, followed by Study 1 including item selection, exploration of a factorial structure, and an initial construct validation, Study 2 including factorial and construct validation, as well as Study 3 including confirmatory factor analysis and further construct validation. For an overview of the development and validation process of the EFHAQ see Supplemental Material Figure S1.

### Pre-study - Item generation and scale development

The Pre-study aimed to develop a first item set assessing EFs based on the model of Miyake and colleagues^5,4^ between May and October 2022. The proposed questionnaire was developed following recommendations from various authors^41,42^. The item generation and scale development (deductive scale development) included the following steps: (1) defining the construct and its subdomains, (2) item development, (3) assessment of content validity of the developed items, (4) defining an answering scale, and (5) conducting cognitive interviews (think aloud method) to refine the items.

In a first step, the construct and its facets were defined based on a literature review. Then, two experts jointly developed a catalog of 65 items (see Supplemental Material), by review of existing scales (e.g., ADEXI^33^, DEX^23,34^, EFI^29^ as well as generating new items; in total, 22 items for updating, 20 items for inhibition, and 23 items for shifting resulted from this first step. Each item clearly represented one of the three EF domains by Miyake et al.^4^ with regard to daily functioning in different contexts (e.g., health behavior, academic achievement, social functioning). After checking these items with regard to clarity, redundancies, and linguistic aspects by an expert in questionnaire development, some of the items were rephrased or dismissed and 16 items for updating, 18 items for inhibition, and 15 items for shifting remained. Subsequently, the remaining items were independently assessed by two experts with regard to content validity, in order to ensure that (1) each item clearly represents one of the three EF domains with regard to daily functioning in different contexts, and (2) the breadth of each EF domain is covered with the respective items. As this check showed that some aspects of the EF domains updating and inhibition were underrepresented, new items regarding these specific aspects were generated. The answering scale of all items ranged from 1 - *never*, 2 - *very rarely*, 3 - *sometimes*, 4 - *often*, 5 - *very often* to 6 – *always*. These items underwent five cognitive interviews with healthy adults using the think aloud method (concurrent session; i.e., participants verbalized their thoughts while reading and answering the questionnaire^43^ and items were refined with regard to clarity and conciseness.

Based on a rule of thumb^44^, it was aimed to generate approximately three times as many items as the questionnaire should comprise in its final version, as it was expected that the statistical analysis (inductive scale development) would substantially reduce the initial item set. Therefore, 55 items were developed (18 items for updating, 22 items for inhibition, and 15 items for shifting) in this Pre-study, which show adequate content validity and clarity in language.

### Study 1 – Item selection, exploration of factorial structure, and construct validation

Study 1 was conducted to evaluate the initially developed item set of the Pre-study based on established item statistics and explore the factorial structure of the questionnaire. Furthermore, we aim to refine the questionnaire and to provide preliminary values of reliability and construct validity for its potential latent factors.

## Methods

### Participants

Participants were recruited via flyers, social media, and personal contacts. Participation was voluntary and without compensation, except for students from MSH Medical School Hamburg, who could earn course credits for their participation. The study fully conformed to the Declaration of Helsinki and the ethics guidelines of the German Psychological Society. The study was positively voted for by the local ethics committee (reference number: MSH-2024/379).

Overall, *N* = 554 participants completed the questionnaire. The participants’ mean age was 23.19 years (*SD* = 4.8; range 18 to 60 years), and 74.2% were female (*n* = 411). The sample was highly educated with the majority of participants (*N* = 535; 96.6%) having an university-entrance diploma (‘Abitur’).

## Measures

Data on the items generated in the Pre-study and other measures were collected via an online questionnaire between February and June 2023 based on the software Enterprise Feedback Suite (EFS; by Tivian).

### EF questionnaire

The 55 EF items from the Pre-study were presented in a randomly mixed order. Items were preceded by the following instruction: “Below you will find a series of statements that relate to situations in your everyday life, e.g., situations at work, school, university, and family life. Please read the instructions and statements carefully and indicate how often you experience this yourself.” The possible answers range from 1 -* never*, 2 - *very rarely*, 3 - *sometimes*, 4 - *often*, 5 - *very often* to 6 - *always*. The subscales that are created in the process of Study 1 were calculated by the mean value of the items loading on a respective factor and higher mean values on a subscale represent better executive functioning.

### Validation scales

As performance-based tasks of EFs were shown to be hardly associated with subjective measures of EFs, we refrained from using these tests for the validation of our questionnaire. However, as we expect EFs to be theoretically intertwined with the concept of self-regulation^3^, and as research implicates frontal brain systems (i.e., executive functioning) to be related to self-regulatory processes^45^, established standardized self-regulation scales were selected for an initial and preliminary convergent construct validation:

#### Trait self-control

Self-control as a personality trait is relatively stable over time and is correlated with a wide variety of life outcomes. As the core of self-control is resisting temptations and resisting acting impulsively, trait self-control should be positively correlated with the inhibition aspect of EFs but also with updating and shifting. Trait self-control was measured with a short German version of the Self-Control Scale of Tangney et al.^46,47^.

#### Perceived general self-efficacy

General self-efficacy is the belief in one’s capabilities to successfully carry out a behavior or a course of action^48^. It is the confidence to be able to control behavior, exert an influence over the environment, and stay motivated in the pursuit of goals. Perceived self-efficacy facilitates self-regulation in terms of goal setting, investment of effort, persistence in the presence of barriers, and the recovery from setbacks. Bates, Pawlak, Tonigan, and Buckman^49^ as well as McAuley et al.^50^ found that self-efficacy mediated the effect of EF on substance abuse and physical activity behavior, respectively. Therefore, self-efficacy should be positively correlated with the EF scores. Perceived general self-efficacy was measured with the General Self-efficacy Scale by Schwarzer and Jerusalem^51^.

#### Procrastination

Procrastination is the intentional delay at the beginning or completion of important and timely activities. Procrastination can be seen as a failure in self-regulation, and procrastinators, compared to non-procrastinators, have a reduced ability to resist (social) temptations, pleasurable activities, and immediate rewards^52^, and, consequently, several EF domains (e.g., inhibition, updating) were shown to be significant predictors of procrastination^52^. Therefore, we assume that procrastination should be negatively correlated with the three EF domains. Procrastination was assessed with a short form of the General Procrastination Scale by Klingsieck and Fries^53^.

### Statistics

We conducted exploratory factor analyses (EFA) with SPSS 27^®^ using principal axis factoring with oblique Promax rotation (reflecting the assumption of intercorrelated latent factors). Non-fitting items were removed based on the following criteria^54^: factor loading < 0.40, cross-loading > 0.30, communality < 0.30, corrected item-scale correlation < 0.30, and the need for at least three items per factor. There were no missing values for the EF items. Skewness and kurtosis of all items were below the critical thresholds of 2 and 7^55^. Bivariate correlations were used to analyse associations between the EF subscales and the validation constructs.

## Results

First, we checked if, in our EF questionnaire, the requirements for EFA in this sample were fulfilled. The results revealed that the data were suitable for conducting an EFA (Kaiser-Meyer-Olkin = 0.91, Bartlett’s test of sphericity χ2(1485) = 12,063.23, *p* < .01). In a first step, we prespecified three factors for the analysis according to the model of Miyake et al.^5,4^. However, the assumed factorial structure did not fit the data. The items initially hypothesized to represent the updating factor were found to load on two factors and were mixed up with the items that were hypothesized to represent the shifting factor. Therefore, in a second step we performed an EFA, using the Kaiser-criterion (eigenvalue > 1) that yielded 13 factors with eigenvalues greater than one. We additionally performed a Velicer’s minimum average partial (MAP) test^56^ which suggested five factors. Additionally, we conducted a parallel analysis using the SPSS syntax from O’Connor^56^ and the parallel analysis suggested 7 factors^57^.

In a third step, we examined the latent factor solutions (5 vs. 7 vs. 13 factors) regarding their plausibility. As the 13-factor and the 7-factor solution contained several factors with less than 3 items, with factor loadings below 0.40, and as we aimed to develop a time efficient instrument, we decided to continue the analysis with the 5-factor solution. Using the initial 5-factor solution, items were removed step by step based on the following criteria: factor loading < 0.40, cross-loading > 0.30, communality < 0.30. In the first round of EFA, 23 items with communalities < 0.30 and/or main loadings < 0.40 were removed. Five further items were removed in the second round (factor loading < 0.40, cross-loading > 0.30, communality < 0.30). 

Forcing the items on the 3-factor solution suggested by Miyake has not produced a convincing pattern as many items did not load onto the factor they were expected to load on, leading to non-plausible latent factors in terms of content. In case of the 5-factor solution, we were able to identify latent variables (i.e., we were able to assign a meaningful label to the underlying factors) which reflect the EF domains inhibition, updating, shifting even though updating and shifting split up into two factors each.

Finally, five factors (we label these factors EF subscales despite their inconsistency with the unity / diversity framework of Miyake et al., 2000) with 27 items (see Table 1) were extracted: *flexibility* (10 items, eigenvalue = 5.31), *focusing* (7 items, eigenvalue = 4.66); *inhibition* (4 items, eigenvalue = 3.17), *adaptation* (3 items, eigenvalue = 3.23), and *weighing up* (3 items, eigenvalue = 2.47).

**Table 1:** Means, standard deviations, item scale correlation and factor loadings of the Executive Functions in Healthy Adults Questionnaire for Studies 1, 2, and 3.

Factor		Item^1^	Sample								
			Study 1 (*n* = 554)			Study 2 (*n* = 411)			Study 3 (*n* = 609)		
			*M* (*SD*)	*r*_*it*_	*a*	*M* (*SD*)	*r*_*it*_	*a*	*M* (*SD*)	*r*_*it*short_	*a* ^2^
flexibility α_1_ = 0.85 α_2_ = 0.81 α_2short_ = 0.67 α_3short_ = 0.77	**[1]**	**When opportunities to achieve something suddenly arise**,** I take them without hesitation.**	3.86 (1.01)	0.52	0.74	4.01 (0.96)	0.46	0.74	4.06 (1.04)	0.69	0.83
	**[2]**	**When a good chance to reach a goal spontaneously comes up**,** I take it.**	4.15 (0.99)	0.59	0.72	4.26 (0.91)	0.48	0.73	4.27 (1.03)	0.68	0.82
	[3]	When I have a plan, nothing can stop me from implementing it.	3.92 (1.02)	0.56	0.71	3.88 (1.01)	0.46	0.51	3.86 (1.10)	0.49	-
	**[4]**	**If one of my plans does not work**,** I immediately take another approach to reach my goal.**	3.80 (1.04)	0.58	0.62	3.75 (98)	0.50	0.57	3.89 (1.07)	0.49	0.57
	[5]	If a solution turns out to be unhelpful, I quickly find an alternative.	3.90 (0.93)	0.59	0.55	3.84 (0.87)	0.61	0.64	3.98 (0.99)	0.64	-
	[6]	I find it very easy to generate different solutions to a problem.	3.94 (1.04)	0.56	0.54	3.89 (1.02)	0.47	0.41	4.02 (1.12)	0.50	-
	[7]	When obstacles suddenly stand in the way of achieving a goal, I simply work around them.	3.73 (0.90)	0.54	0.52	3.70 (0.84)	0.58	0.51	3.76 (0.99)	0.59	-
	[8]	I find it easy to integrate different pieces of information to make a decision.	4.08 (1.06)	0.54	0.51	3.84 (1.05)	0.43	0.26	4.05 (1.11)	0.45	-
	[9]	I take in new information quickly and use it in a goal-directed way.	3.95 (0.93)	0.59	0.51	3.89 (0.89)	0.53	0.28	4.09 (0.98)	0.63	-
	[10]	I can easily understand new instructions and put them into practice right away.	4.19 (0.93)	0.55	0.48	4.11 (0.88)	0.46	< 0.20	4.21 (0.96)	0.55	-
focusing α_1_ = 0.81 α_2_ = 0.83 α_2short_ = 0.72 α_3short_ = 0.73	**[1]**	**I can focus my attention completely on one task for a long period of time.**	3.56 (1.08)	0.59	0.65	3.39 (1.06)	0.64	0.66	3.74 (1.12)	0.57	0.73
	**[2]**	**I can concentrate well on a task despite external distractions (e.g.**,** loud noises).**	3.13 (1.18)	0.55	0.65	3.38 (1.18)	0.62	0.67	3.46 (1.15)	0.49	0.60
	[3]	I am good at blocking out distracting thoughts that that prevent me from completing a task.	3.17 (1.06)	0.60	0.64	3.08 (0.99)	0.57	0.56	3.38 (1.06)	0.58	-
	[4]	My attention span is rather short.*	3.84 (1.09)	0.55	0.62	3.79 (1.10)	0.56	0.60	3.93 (1.15)	0.57	-
	**[5]**	**When I get distracted from a task (e.g.**,** by my phone or by other people)**,** I can tune it out and get back to the task immediately.**	3.34 (0.99)	0.58	0.62	3.30 (0.97)	0.66	0.74	3.48 (1.12)	0.59	0.74
	[6]	External distractions (e.g., from my phone or from other people) distract me from my work.*	3.41 (1.09)	0.53	0.58	3.53 (0.97)	0.64	0.80	3.69 (1.06)	0.56	-
	[7]	I need complete silence to stay focused on a task.*	3.39 (1.29)	0.41	0.57	3.42 (1.23)	0.41	0.58	3.46 (1.23)	0.37	-
inhibition α_1_ = 0.82 α_2_ = 0.70 α_2short_ = 0.69 α_3short_ = 0.72	**[1]**	**I find it difficult to suppress bad habits (e.g.**,** drinking too much alcohol**,** eating sweets**, or raising my voice during arguments).*	3.99 (1.17)	0.72	0.87	4.09 (1.05)	0.60	0.73	3.97 (1.11)	0.57	0.76
	**[2]**	**I do things that are bad for me when they are enjoyable (e.g.**, drinking too much alcohol or eating sweets).*	3.67 (1.16)	0.69	0.80	3.56 (1.01)	0.56	0.77	3.65 (1.12)	0.56	0.71
	**[3]**	**I cannot stop myself from doing something even though I know it is wrong (e.g.**, drinking too much alcohol).*	3.97 (1.18)	0.68	0.73	4.26 (1.11)	0.41	0.49	4.20 (1.16)	0.47	0.58
	[4]	I am good at resisting temptations.	3.49 (1.12)	0.48	0.41	3.46 (0.98)	0.40	0.42	3.54 (1.03)	0.46	-
adaptation α_1_ = 0.79 α_2_ = 0.82 α_2short_ = 0.82 α_3short_ = 0.78	**[1]**	**I can respond flexibly to changes.**	3.81 (1.03)	0.68	0.78	3.98 (1.00)	0.71	0.87	4.10 (1.11)	0.72	0.88
	**[2]**	**I can easily adjust to changes (e.g.**,** a change of plans).**	3.76 (1.08)	0.65	0.71	3.72 (1.13)	0.73	0.83	3.99 (1.15)	0.70	0.87
	**[3]**	I find it difficult to adapt spontaneously to changes*	3.99 (1.16)	0.55	0.61	4.26 (0.97)	0.57	0.71	4.14 (1.13)	0.46	0.49
weighing up α_1_ = 0.73 α_2_ = 0.78 α_2short_ = 0.78 α_3short_ = 0.78	**[1]**	**I carefully consider all options before taking action.**	4.12 (1.15)	0.58	0.75	3.99 (1.11)	0.66	0.82	3.95 (1.09)	0.54	0.60
	**[2]**	I act without having considered all alternatives.*	4.60 (1.03)	0.59	0.71	4.46 (1.04)	0.60	0.67	4.35 (1.08)	0.66	0.82
	**[3]**	I act without thinking and do the first thing that comes to mind.*	4.56 (1.00)	0.51	0.64	4.63 (0.96)	0.59	0.72	4.47 (1.06)	0.64	0.79

M – means, SD – standard deviations, r_it_ – corrected item-scale correlations, a – factor loadings of EFA, a^2^ – factor loadings of CFA, bold items – final item set, short – shortened 15 item version.

^1^Not validated English version of the items translated by the authors. The German version of the EF-Scale is shown in Table S1.

^*^inversed items.

The factors accounted for 46.17% of the variance, and the five factors are interpretable and meaningful. That is, the factor *flexibility* consists of items reflecting the ability to spontaneously seize an opportunity that arises while striving to achieve a goal or find a solution for a problem. *Focusing* covers being able to stay focused and being concentrated on an activity during the presence of potential distractions (e.g., noise, other persons). The factor *inhibition* captures the ability to inhibit unfavorable behavior (such as drinking too much alcohol) in the presence of behavioral stimuli or their absence. *Adaptation* covers items that reflect the ability to adapt behaviors to sudden unfavorable changes in the environment. *Weighing up* comprises items reflecting the ability to think through alternatives before starting to act.

Table 1 shows the internal consistencies and the associated items with means, standard deviations, item-scale correlations, and factor loadings. Please note, the items in Table 1 are not validated, they are translated into English from the original German version (see Supplemental Material Table S1). The factor loadings range from 0.41 to 0.87. All five factors showed acceptable to good internal consistencies ranging from 0.73 to 0.85.

The five EF subscales showed low to moderate inter-correlations (Table 2). These correlations are in line with the unity/diversity framework of EFs (Miyake et al., 2000), which assumes that there is a pattern of intercorrelation (unity) among EF domains, which are also separable (diversity) domains of EFs.

**Table 2:** Correlations between the subscales of the Executive Functions in Healthy Adults Questionnaire, trait self-control, perceived general self-efficacy, procrastination in Study 1.

	1	2	3	4	5
1 - EF flexibility					
2 - EF focusing	**0.44**				
3 - EF inhibition	**0.26**	**0.34**			
4 - EF adaptation	**0.45**	**0.38**	**0.16**		
5 - EF weighing up	**0.14**	**0.15**	**0.35**	*− 0.08*	
6 - self-control	**0.38**	**0.54**	**0.70**	**0.19**	**0.45**
7 - self-efficacy	**0.73**	**0.43**	**0.28**	**0.52**	*0.07*
8 - procrastination	**− 0.25**	**− 0.45**	**− 0.43**	*− 0.06*	**− 0.27**

Notes: bold: *p* < .05; italic: *p* > .05.

Overall, the five EF subscales showed hypothesized correlations (low to high) with trait self-control, perceived general self-efficacy, and procrastination (Table 2). Only, the subscale adaptation was not correlated with procrastination and the subscale weighing up was not associated with general self-efficacy. These results can be interpreted as preliminary indications for convergent construct validity of the newly developed subscales.

## Discussion

Study 1 aimed to systematically evaluate the initially developed items based on item statistics and to examine the factorial structure of the EFHAQ. Based on these evaluations, the questionnaire was reduced to 27 items which split up into five factors (i.e., EF subscales). The subscale inhibition was in line with the theoretical assumptions, while the factor updating (focusing and weighing up) as well as the factor shifting (flexibility and adaptation) split up into two subscales. Preliminary validation checks revealed the expected associations of the five EF subscales with associated constructs. Internal consistency was acceptable to good. However, due to the somewhat ambiguous factor structure in Study 1 we decided to conduct a second EFA in Study 2 to eliminate further inappropriate items and to achieve a sound factor solution. Even though we believe that the observed deviation from the 3-factor structure is still within the scope of the model of Miyake (the factors inhibition, updating and shifting are implicitly represented, even though the factors updating and shifting split into two underlying factors each) and as we have not intended to test the original theoretical model of Miyake we changed for this purpose from a theory-driven (deductive) to a data-driven (inductive) questionnaire development approach at this point.

### Study 2 – Factorial and construct validation

This study aimed to further explore the factorial structure of EFHAQ that has been found in Study 1. As the initially expected three-factor solution was not found (instead a five-factor solution appeared) and as the number of items was substantially reduced in Study 1 (from 55 to 27), we decided to conduct a second EFA based on the reduced item set with an independent participant sample. We aimed to eliminate further non-fitting items to achieve a stable factorial structure and a time efficient questionnaire. Additionally, we examined associations with relevant variables (related constructs such as impulsivity) to provide further hints for construct validity as well as first results on criterion validity.

## Methods

### Participants

Participants were recruited via flyers, social media, and personal contacts at different German universities (e.g., MSH Medical School Hamburg, University of Frankfurt, University of Konstanz, Karlsruher Institute of Technology). Participation was voluntary and without compensation, except for students from MSH Medical School Hamburg, who could earn course credits for participating in the study. The study fully conformed to the Declaration of Helsinki and the ethics guidelines of the German Psychological Society. The study was positively voted for by the local ethics committee (reference number: MSH-2024/379). Due to technical issues, no biographical data (i.e., gender, age) was recorded for this study. (We will elaborate on this issue in more detail in later discussion sections.) However, overall, *N* = 411 participants completed the questionnaire between March and July 2024.

## Measures

### EF questionnaire

The remaining 27 items of Study 1 were presented in a randomly mixed order. All other details of the EF questionnaire remained identical to this study.

### Validation measures

As we expected EFs to be associated with different aspects of self-regulation, various self-regulation scales as well as personality traits were selected for construct validation. These constructs differed from Study 1 to provide further validity information:

### Construct validity

#### Impulsivity

Shapiro^58^ defined impulsivity as the tendency toward nonreflective cognition or the lack of controlled analytic information processing. Results of empirical studies revealed that impulsivity and EFs might be slightly negatively associated^59^. Therefore, we assume that better scores in subjectively reported EFs are associated with lower impulsivity. Impulsivity was assessed with a German version of the Brief UPPS Impulsive Behavior Scales^59^ including the four subscales *urgency*, *lack of premeditation*, *lack of perseverance*, and *sensation seeking*.

#### Grit

Grit is “…defined as trait-level perseverance and passion for long-term goals…”^60^ (p. 166). It comprises the capacity to sustain *interest* and *effort* in goals over months or even longer. Grit was shown to be positively associated with EFs and to mediate the association between EFs and well-being^61^. Consequently, we assumed that grit should be positively associated with the EF domains of our questionnaire. Grit was measured with a German adaptation of the 12-Item Grit Scale^62^ including the subscales *consistency of interests* and *perseverance of effort*.

#### Conscientiousness

It is assumed that personality traits shape how individuals make use of and control their cognitive abilities^63^. Associations between personality traits and EFs have been found based on various measures of EFs, including self-report questionnaires. Derived from these studies, we expected that conscientiousness is (positively) moderately associated with the EF domains. Conscientiousness was measured with the respective subscale of the German version of the Short Version of the Big Five Inventory^64^.

### Concurrent criterion validity

An ongoing health behavior, i.e., physical activity behavior, was chosen and measured concurrently with the EFs, as this behavior is thought to be the result of successful self-regulation and investing effort^65^. Physical activity was measured with four items derived from the International Physical Activity Questionnaire (IPAQ short form^66^. The moderate-to-vigorous physical activity (MVPA) score was calculated by multiplying frequency of physical activity (during the last 7 days) with average duration per occasion in minutes for vigorous and moderate physical activities respectively. Finally, these values were summed up resulting in the MVPA score.

### Statistics

We conducted EFA with SPSS 27^®^ using principal axis factoring with oblique Promax rotation. Non-fitting items were removed according to the criteria mentioned in Study 1. Aiming to examine construct validity, we conducted bivariate correlation analyses between the EF subscales and the above-mentioned constructs.

## Results

The structure with the five latent EF factors (flexibility, focusing, inhibition, adaptation, weighing up) has been replicated with the sample of Study 2. Internal consistencies of these factors and the associated items with means, standard deviations, item-scale correlations, and factor loadings are shown in Table 1. As our aim was to provide a stable time efficient instrument, we eliminated 7 further items from the flexibility subscale, 4 items from the focusing subscale, and 1 item from the inhibition subscale. The decision was based on factor loadings, item-scale correlation, and cross loadings on other than the assumed factor. Internal consistencies (Cronbach`s alpha) were acceptable for each of the five EF subscales.

As assumed, we found positive correlations between the EF subscales and several related constructs. We performed bivariate correlation analyses with the EF subscales of the long (27 items; Table 3) and the shortened (15 items; Table S2) questionnaire and found mostly comparable correlation patterns.

**Table 3:** Correlations between the subscales of the Executive Functions in Healthy Adults Questionnaire (27 items), and relevant constructs in Study 2.

	1	2	3	4	5
1 - EF flexibility					
2 - EF focusing	**0.35**				
3 - EF inhibition	**0.21**	**0.31**			
4 - EF adaptation	**0.31**	**0.29**	*0.09*		
5 - EF weighing up	**0.11**	**0.18**	**0.33**	*-0.07*	
6 - conscientiousness	**0.27**	**0.39**	**0.35**	*0.05*	**0.36**
7 - MVPA	**0.13**	**0.13**	**0.15**	**0.13**	*0.08*
8 – impulsivity urgency	**0.27**	**0.37**	**0.55**	**0.22**	**0.33**
9 – impulsivity lack of premeditation	**0.12**	**0.15**	**0.34**	**-0.12**	**0.69**
10 – impulsivity lack of perseverance	**0.41**	**0.44**	**0.41**	**0.22**	**0.** **27**
11 – impulsivity sensation seeking	**-0** **.17**	*-0.08*	*0.04*	**-0.30**	**0.** **1** **2**
12 – grit consistency of interests	**0.25**	**0.40**	**0.39**	**0.19**	**0.** **29**
13 – grit perseverance of effort	**0.44**	**0.41**	**0.38**	**0.21**	**0.** **3** **0**

Notes: bold: *p* < .05; italic: *p* > .05.

The EF subscales were significantly positively associated with *conscientiousness* (the better the EF the higher conscientiousness) as well as with the two subscales of grit *consistency of interests* and *perseverance of effort* (the better the EF the higher grit). There were negative associations between the EF subscales and three subscales of impulsivity *urgency*, *lack of premeditation*, and *lack of perseverance* (the better the EF the lower impulsivity) as well as lower and mainly negative correlations between the EF subscales and the impulsivity subscale *sensation seeking.* Physical activity was chosen to provide initial criterion validity. The correlation of physical activity and the EF subscales were slightly positive.

## Discussion

Study 2 aimed to further explore the factorial structure of the set of 27 items and to provide further results on validity of the EFHAQ. The five-factor solution of Study 1 was confirmed in Study 2 indicating a stable factor solution. Driven by the statistical analyses, the item set was further reduced from 27 to 15 items to get a time efficient questionnaire. Internal consistencies were acceptable to good for both versions. The validity checks for the five subscales provided further hints on construct and criterion validity of the subscales and correlation patterns were comparable for the 27 and the 15-item version, indicating adequate test quality also for the shortened version. Based on the quality criterion of test economy, we decided to cross-validate mainly the 15-item version in the next study. Due to technical error, sociodemographic data (i.e., gender, age, education) could not be recorded in Study 2. As data collection was mainly conducted at universities, we assume that sample characteristics of Study 2 were comparable to Study 1. However, we are unable to formally characterize this sample or rule out the possibility that it differed in composition from Study 1. This limitation is critical for the item-reduction process conducted in Study 2 and the 15-item structure should be interpreted with this constraint in mind until replicated in a sample with documented demographics. Because of this issue, Study 3 includes the relevant recordings of sociodemographic data.

### Study 3 - Cross-validation and confirmatory factor analysis

The aim of Study 3 was to cross-validate the findings of Study 2 and to provide further support for the factorial as well as construct validity of the EFHAQ. Furthermore, we examined criterion validity, internal consistency, and test-retest reliability. Therefore, we firstly performed a confirmatory factor analysis using the shortened item set of Study 2. Secondly, we examined associations between the shortened EF subscales and associated constructs and criteria to provide further evidence for the validity of the questionnaire and its subscales. Thirdly, test-retest reliability was assessed after three weeks in a subgroup of the participant sample.

## Methods

### Participants

Participants were recruited via flyers, social media, and personal contacts. Furthermore, 100 participants were recruited via the platform SurveySwap (https://surveyswap.io/). Participation was voluntary and without compensation, except for students from MSH Medical School Hamburg, who could earn course credits for their study participation and participants of SurveySwap, who were compensated with 10 €. The study fully conformed to the Declaration of Helsinki and the ethics guidelines of the German Psychological Society. The study was positively voted for by the local ethics committee (reference number: MSH-2024/379). Participants were invited to fill in a second questionnaire after three weeks to assess test-retest reliability of the EF questionnaire.

Overall, *N* = 609 participants completed the questionnaire between April and July 2025. The participants’ mean age was 30.83 years (*SD* = 12.78; range 18 to 82 years), and 58.3% were female (*n* = 355). The sample is highly educated with the majority of participants (*N* = 396) have a university-entrance diploma (‘Abitur’) or a higher degree (e.g., bachelor or master`s degree). Of these 609 participants, 104 also filled in the retest questionnaire after three weeks.

## Measures

### EF questionnaire

This questionnaire was identical to the 15-item version in Study 2.

### Validation measures

*Construct validity.* As we expected EFs to be intertwined and associated with different aspects of self-regulation, various self-regulation scales as well as personality traits were selected for construct validation. To provide further information on criterion validity, well-being, and satisfaction with life scales were included:

#### Big Five personality traits

Big Five personality traits were partly shown to be correlated with EFs in previous studies. Derived from these studies, we expected that conscientiousness (positively) and neuroticism (negatively) are moderately associated with the EF subscales, while extraversion, openness, and agreeableness should show lower positive correlations. Big Five personality traits were measured with the Big Five Inventory 10^67^.

#### Trait self-control

 This questionnaire was identical to the one in Study 1.

#### Perceived general self-efficacy

This questionnaire was identical to the one in Study 1.

#### Procrastination

This questionnaire was identical to the one in Study 2.

#### Grit

 This questionnaire was identical to the one in Study 2.

#### Concurrent criterion validity

For assessing criterion validity, constructs were chosen that have been repeatedly shown to be a result of successful self-regulation.

#### Physical activity behavior

 was recorded identical to Study 2.

According to Diener, Oishi, and Tay^68^*subjective well-being* contains (1) affective evaluations of a person’s positive and negative emotions and (2) cognitive evaluations of global life satisfaction. It was shown that EFs are associated with subjective well-being. EF and subjective well-being seem to be located in similar brain regions.

#### Well-being (affective evaluation)

Short, Mazmanian, Oinonen, and Mushquash^69^ found significant correlations (moderate to strong) between EFs and positive and negative affect and EFs were shown to be associated with emotion regulation strategies^70^. Accordingly, we expected positive relationships between EFs and affective well-being. The affective evaluation of well-being was assessed with the WHO-5 well-being index^71,72^.

#### Satisfaction with life (cognitive evaluation)

Life satisfaction is the cognitive component of well-being and is based on a judgmental process. It is a person`s global judgement of the quality of their lives based on a comparison of one`s life circumstances with a unique personal set of criteria^68^. As better EFs seem to be associated with better self-regulation, people with better EFs might be better in achieving relevant life goals and positive outcomes. In line with this assumption, EFs were associated with life satisfaction^73,74^. Accordingly, better subjective EFs should be positively correlated with satisfaction with life. Satisfaction with life was assessed with a German version of the satisfaction with life scale (SWLS^75,76^).

#### Statistics

Confirmatory factor analyses were performed with AMOS 27^®^ using the maximum-likelihood method with the sample of Study 3. The commonly recommended fit indices χ2/df, CFI, SRMR, and RMSEA were used to assess the goodness of fit. A good fit is indicated by 0 ≤ χ2/df ≤ 2, 0.97 ≤ CFI ≤ 1, 0 ≤ SRMR ≤ 0.05 and RMSEA ≤ 0.05, while values 2 < χ2/df ≤ 3, 0.95 ≤ CFI < 0.97, 0.05 < SRMR ≤ 0.10 and 0.05 < RMSEA ≤ 0.08 indicate an acceptable fit^77^. Construct as well as criterion validity was tested with bivariate correlations between the shortened EF subscales and the above-mentioned constructs. Further bivariate correlations were used to examine test-retest reliability.

## Results

**Fig. 1:**
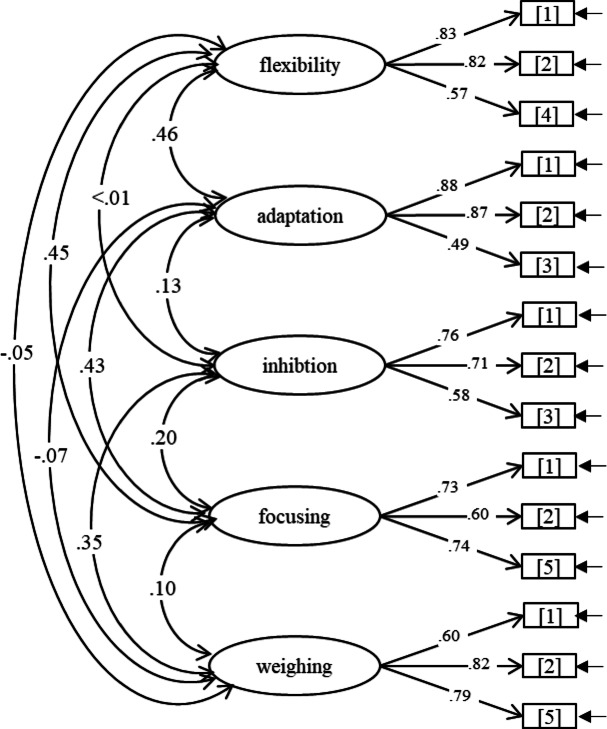
Measurement model of the executive functions in healthy adults questionnaire based on CFA in Study 3.

Internal consistencies, means, standard deviations, item-scale correlations, and factor loadings for the EF subscales are shown in Table 1. Figure 1 depicts the final measurement model. According to the fit indices RMSEA and SRMR the model demonstrated an acceptable fit (*χ*^2^ = 256.57, df = 80, *p* < .001; *χ*^2^/df = 3.21; CFI = 0.94; SRMR = 0.06; RMSEA = 0.06, CI 0.05/0.07). However, the other fit indices (CFI / *χ*^2^/df) were slightly below / above the thresholds. Figure S2 depicts an alternative measurement model with two second-order factors (shifting and updating) and one first-order factor (inhibition) that corresponds to the assumption of Miyake and Friedman^5^. The fit indices were as follows: *χ*^2^ = 358.21, df = 85, *p* < .001; *χ*^2^/df = 4.21; CFI = 0.91; SRMR = 0.09; RMSEA = 0.07, CI 0.07/0.08.

Internal consistencies (Cronbach`s alpha) were acceptable for all subscales. The results for test-retest reliability based on bivariate correlations (*N* = 104) are between 0.64 und 0.72, (r_flexibility_ = 0.70, *p* < .001; r_focusing_ = 0.70, *p*<.001; r_inhibition_ = 0.64, *p*<.001; r_adaptation_ = 0.70, p <. 0.001; r_weighing up_ = 0.72, *p* < .001) and therefore partly below the threshold of 0.70 for acceptable reliability.

As expected, the five subscales of the EF questionnaire correlated significantly with the assessed validation constructs. With regard to the Big Five personality traits, we found low to moderate correlations in the supposed direction with *conscientiousness* (except for the subscale adaptation), *neuroticism* (except for the subscale weighing up), and *extraversion* (except for the subscales inhibition and weighing up), as well as low correlations with *openness* (except for the subscale focusing). *Agreeableness* only slightly correlated with focusing and adaptation (Table 4).

**Table 4:** Correlations between the *shortened* subscales of the Executive Functions in Healthy Adults Questionnaire (15 items) and relevant constructs in Study 3.

	1	2	3	4	5
1 - EF flexibility					
2 - EF focusing	**0.37**				
3 - EF inhibition	*0.01*	**0.13**			
4 - EF adaptation	**0.38**	**0.30**	**0.14**		
5 - EF weighing up	*− 0.02*	**0.10**	**0.24**	*− 0.01*	
6 – extraversion	**0.30**	**0.17**	*0.06*	**0.30**	*− 0.07*
7 – agreeableness	*0.07*	**0.12**	*0.02*	**0.15**	*− 0.06*
8 – conscientiousness	**0.28**	**0.28**	**0.33**	*0.06*	**0.28**
9 – neuroticism	**− 0.31**	**− 0.30**	**− 0.20**	**− 0.39**	*− 0.10*
10 – openness	**0.16**	*0.05*	**0.13**	**0.14**	**0.12**
11 – self-control	**0.17**	**0.34**	**0.58**	**0.19**	**0.39**
12 – self-efficacy	**0.61**	**0.41**	*0.10*	**0.52**	*0.10*
13 – procrastination	**− 0.23**	**− 0.36**	**− 0.30**	*− 0.02*	**− 0.13**
14 – grit consistency of interest	*0.09*	**0.16**	**0.31**	*0.06*	**0.24**
15 – grit perseverance of effort	**0.50**	**0.34**	**0.13**	**0.27**	**0.20**
16 – satisfaction with life	**0.39**	**0.33**	**0.13**	**0.22**	**0.13**
17 – WHO-5 Well-Being Index	**0.31**	**0.27**	**0.12**	**0.17**	*0.05*
18 – MVPA	*0.04*	*0.01*	*− 0.02*	*0.05*	*− 0.03*

Notes: bold: *p* < .05; italic: *p* > .05.

We also found the expected correlations with *trait self-control* (strongest correlation with inhibition), *self-efficacy* (except for the subscales inhibition and weighing up), *procrastination* (except for the subscale adaptation) as well as for the two subscales of *grit*, whereat these correlations are more pronounced for *perseverance of effort* compared to *consistency of interests.*

With regard to the criterion validity, the association between the EF subscales and satisfaction with life as well as affective well-being have been confirmed. These criteria were mainly associated with flexibility and focusing. However, the correlations with MVPA were not significant (Table 4).

Descriptive statistics for the subscales of the EFHAQ in Study 3 are reported in Table S3.

## Discussion

The aim of Study 3 was to cross-validate the factorial structure of Study 2 based on the reduced 15-item set in a confirmatory factor analysis in an independent sample with recorded biographical data. Overall, the five-factor solution fitted the data well and the factor structure was shown to be stable. Internal consistencies were acceptable, while test-retest reliability was below the accepted threshold in one subscale. These findings do not fully support the expectations and suggest that the stability of the EFHAQ may be more limited than we expected. The pattern of correlations supported the construct and criterion validity of the developed EF questionnaire and its subscales. Therefore, the questionnaire seems to be a sufficiently reliable, valid, and time efficient tool that can be used to study research questions associated with EFs. However, as the model fit was adequate but not strong, future studies should provide further examinations of the factor structure to refine the scales and improve the fit indices (e.g., through item refinement) where applicable.

## General discussion

The aim of the study was to develop and validate an ecologically valid and time-efficient self-report questionnaire for healthy adults assessing EFs in the context of the unity/diversity framework of Miyake and colleagues^5,4^(i.e., inhibition, updating, shifting). With the EFHAQ, we aimed to provide a time-efficient open-access tool for healthy adults, which is applicable for research across various contexts such as health behavior, academic achievement or social functioning.

Based on item and factor analyses, the initial item pool of 55 items was reduced to 15 items across the three studies. These items did not split into the expected three-factor structure of the unity/diversity framework of Miyake and colleagues. Instead, the factors *shifting* and *updating* split into two subscales, each resulting in a five-factor structure. Each factor (i.e., subscale) is represented by three items. The factorial structure of EFs regarding their unity and diversity based on performance-based tasks is still debated and seems to be unclear. As our items represent EFs how they manifest in everyday life the differences regarding the factor structure (3 vs. 5 factors) might be explained by the different focus of the measure compared to performance-based tasks. Our items represent the multidimensional, context-dependent nature of real-world decision-making while performance-based tasks often break down an integrated system into artificial components and mainly measure efficiency in decision-making. The five subscales are also consistent with self-regulation theory. The subscale *flexibility* reflects the ability to spontaneously seize an opportunity that arises while striving to achieve a goal or identify a solution for a problem. *Focusing* covers being able to stay focused and being concentrated on an activity during the presence of potential distractions (e.g., noise, other persons). The factor *inhibition* captures the ability to inhibit unfavorable behavior (such as drinking too much alcohol) in the presence of behavioral stimuli or their absence. *Adaptation* covers items that reflect the ability to adapt behaviors to sudden unfavorable changes in the environment. *Weighing up* comprises items reflecting the ability to think through alternatives before starting to act. The five subscales represent relevant higher-order cognitive functions on which self-regulatory strategies and skills, such as planning, goal setting, or monitoring, are based.

Reliability was examined based on (1) internal consistency (Cronbach`s alpha) and (2) test-retest reliability (stability). Internal consistencies of the five subscales were acceptable to good in the three studies. Regarding test-retest reliability, the correlations between the two points of measurement (three weeks apart) were satisfactory except for the subscale inhibition. Inhibition showed a test-retest reliability below the conventional threshold of 0.70. This subscale should be re-evaluated regarding test-retest reliability in future studies. However, even though EFs are thought to be stable over time, individuals might evaluate their EFs based on current states such as mental fatigue, tiredness, exhaustion, or mood, implying that EFs also share state-like aspects^28^. This might explain why test-retest reliability of EFs was not as good as expected and that the stability of the construct was modest, as shown in Study 3. Furthermore, each subscale is represented by three items, which fulfils the requirement of a time-efficient measurement of EFs. However, the reliability of the EFHAQ can be explained by this smaller number of items per subscale.

The validity of the EFHAQ was examined based on various facets of validity: (1) content validity, (2) factorial validity, (3) construct validity, and (4) criterion validity. Firstly, content validity was checked independently by two experts in the field of EFs and self-regulation. These initial checks resulted in items with high content validity, even though it must be examined if the remaining 15 items still adequately capture the breadth and ecological complexity of EF. Secondly, factorial validity was consistently confirmed across three studies. The five-factor structure proved to be stable across three large independent samples and was also reproducible with confirmatory factor analysis. Inter-correlations of the five subscales provided support for the unity/diversity of EFs. Correlation coefficients were small to medium, indicating that the five subscales measure diverse aspects of EFs, while there is also a small to medium overlap between these subscales, implying that they belong to the same EF construct (i.e., unity). Thirdly, construct validity was examined with established standardized questionnaires assessing diverse aspects of self-regulation and personality, such as impulsivity, grit, self-control, Big Five personality traits, or procrastination. The correlations between the five subscales of the EF questionnaire and these self-regulation measures showed the expected correlation patterns across the three validation studies. The five subscales showed low to moderate associations with the self-regulation scales, which means that the EF subscales measure associated but distinctive constructs. The highest correlations of 0.61 and 0.58 were found between the subscales flexibility and self-efficacy and inhibition and self-control . Fourthly, criterion validity was examined based on concurrent assessments of relevant self-regulatory outcomes: Physical activity behavior as a relevant health behavior, as well as the affective and cognitive evaluation of well-being. Low to moderate correlations were shown for affective and cognitive evaluation of subjective well-being, while physical activity behavior was slightly correlated with EFs in Studies 1 and 2 but not in Study 3. The relationship between EF and physical activity is complex and often indirect, involving multiple mediating and moderating processes rather than a simple direct association between EF and physical activity. Therefore, future studies should include assessments of relevant moderators and mediators of the association between EFs and physical activity (e.g., motivation) to further examine the scale`s predictive scope.

### Limitations

While self-report questionnaires are valuable tools for assessing EFs, there are also downsides to using self-report questionnaires. These measures rely on participants’ subjective perceptions and therefore lack objectivity, which may lead to response biases or social desirability, as well as a lack in detecting subtle EF deficits. However, self-report questionnaires are valuable tools for assessing EFs, and as Corneille and Gawronski^30^ argue, the mentioned problems can barely be solved by using performance-based measures.

With only five participants, the cognitive interviewing (think aloud procedure) in the pre-study might not have captured the full range of item interpretation, language comprehension, and response strategies represented in a broad healthy adult population. Future work should address this limitation with a larger and more divers cognitive interviewing sample.

The samples of the three studies contain young, predominantly female, and highly educated individuals and are therefore not representative of the general healthy adult population (note that the specific biographical data were not recorded in Study 2.) Generalization of the results is therefore mainly feasible for younger healthy adult women. Recruiting more diverse samples of healthy adults, representative of the general adult population and testing the psychometric properties of the questionnaire in the future, seems to be crucial.

The construct and criterion validation of the EFHAQ was exclusively based on self-report measures which might have produced a common method bias. The observed associations may be inflated due to shared variance rather than reflecting true construct-level relationships. Therefore, confidence in the validity evidence should be strengthened by including more appropriate objective measures, such as health status (e.g., blood pressure, triglycerides) or academic achievement (e.g., grades), in the validation process. Future studies should use these measures to further assess the validity of the questionnaire.

### Practical implications

Understanding the role of EFs in health behaviors, academic achievement or social functioning is crucial to designing meaningful interventions. Strengthening, for instance, inhibition, flexibility, and focusing abilities may help individuals overcome impulsive behaviors and maintain long-term goals. Therefore, recognizing the relevance of EFs for self-regulation and goal attainment can inform practical strategies across diverse contexts, ultimately improving outcomes for individuals and communities. A complementary use of performance-based tasks and self-report questionnaires might provide valuable and distinct insights in these contexts.

## Conclusions

The developed self-report EFHAQ is a sufficiently reliable and valid tool measuring EFs of healthy adults based on the framework of Miyake and colleagues^5,4^. It offers acceptable psychometric properties for studying individual differences in EFs. With this questionnaire, we provided a time-efficient tool with 15 items and five subscales that can be used to examine research questions in health, social, cognitive, sport or educational psychology. Our findings support the usefulness of the EFHAQ for the most part, even though the evidence for the EFHAQ`s reliability and validity has still to be considered as preliminary and limited. Future studies should aim to improve the model`s fit indices by item refinement and further examine test-retest reliability, discriminant validity as well as prognostic criterion validity of this questionnaire. Furthermore, future research is needed to adapt this scale to other populations such as healthy adolescents or older adults. As EFs might contribute to self-regulatory outcomes in different ways (e.g., as a predictor, moderator, or mediator^3^, a series of research questions can be examined based on this questionnaire measure in the future.

## Supplementary Information

Below is the link to the electronic supplementary material.

Supplementary Material 1

## Data Availability

The authors are willing to share their data, analytics methods, and study materials with other researchers. The material will be available upon request from the corresponding author.

## Declarations

### Open Science

The authors are willing to share their data, analytics methods, and study materials with other researchers. The material will be available upon request from the corresponding author.
