# Supplementary material for: Development and validation of a self-report questionnaire to assess executive functions in healthy adults: the executive functions in healthy adults questionnaire (EFHAQ)
Source: Sci Rep. 2026 Aug 8;16:24536. doi: 10.1038/s41598-026-65768-y (PMC13451202; doi:10.1038/s41598-026-65768-y)
Supplement: Supplementary file 1 [file 41598_2026_65768_MOESM1_ESM.docx]

Figure S_1. Flowchart of the development and validation studies of the Executive Functions in Healthy Adults Questionnaire.

Figure S_2. Alternative measurement model with two second-order factors and one first-order factor based on CFA in Study 3

Table S_1: German version of the Executive Functions in Healthy Adults Questionnaire (Original version).

| **Faktor** |  | **Item** |
| --- | --- | --- |
| Flexibilität | **[1]** | **Wenn sich plötzlich Möglichkeiten bieten, etwas zu erreichen, nutze ich diese, ohne zu zögern.** |
|  | **[2]** | **Wenn sich spontan eine gute Gelegenheit für die Erreichung eines Ziels bietet, dann nutze ich diese.** |
|  | [3] | Wenn ich einen Plan habe, hält mich nichts davon ab, ihn umzusetzen. |
|  | **[4]** | **Wenn einer meiner Pläne nicht funktioniert, gehe ich, ohne zu zögern einen anderen Weg, um doch noch ans Ziel zu kommen.** |
|  | [5] | Wenn sich ein Lösungsweg als nicht hilfreich herausstellt, finde ich umgehend einen anderen. |
|  | [6] | Es fällt mir sehr leicht, verschiedene Lösungen für ein Problem zu entwickeln. |
|  | [7] | Wenn der Erreichung eines Ziels plötzlich Hindernisse im Weg stehen, umgehe ich diese einfach. |
|  | [8] | Es fällt mir sehr leicht, unterschiedliche Informationen zusammenzufassen, um damit eine Entscheidung zu treffen. |
|  | [9] | Neue Informationen nehme ich sofort auf und verarbeite sie zielführend |
|  | [10] | Neue Anweisungen kann ich leicht aufnehmen und direkt umsetzen. |
| Fokussierung | **[1]** | **Ich kann meine Aufmerksamkeit für lange Zeit voll und ganz auf eine Sache konzentrieren.** |
|  | **[2]** | **Ich kann mich trotz äußerer Einflüsse (z.B. laute Geräusche) gut auf eine Aufgabe konzentrieren.** |
|  | [3] | Störende Gedanken, die mich von einer Aufgabe abhalten, kann ich gut ausblenden. |
|  | [4] | Meine Aufmerksamkeitsspanne ist eher kurz.* |
|  | **[5]** | **Wenn ich von einer Aufgabe abgelenkt werde (z.B. vom Handy oder anderen Personen), kann ich das ausblenden und finde sofort wieder zur Aufgabe zurück.** |
|  | [6] | Äußere Einflüsse (z.B. vom Handy oder anderen Personen) halten mich von meiner Arbeit ab.* |
|  | [7] | Ich brauche absolute Ruhe, um konzentriert bei einer Aufgabe zu bleiben.* |
| Inhibition | **[1]** | **Schlechte Gewohnheiten zu unterdrücken (z.B. zu viel Alkohol trinken, Süßigkeiten essen oder im Streit laut werden), fällt mir schwer.*** |
|  | **[2]** | **Ich tue Dinge, die schlecht für mich sind, wenn sie mir Spaß machen (z.B. zu viel Alkohol trinken oder Süßigkeiten essen).*** |
|  | **[3]** | **Ich kann mich selbst nicht davon abhalten, etwas zu tun, obwohl ich weiß, dass es falsch ist (z.B. zu viel Alkohol trinken).*** |
|  | [4] | Ich bin gut darin, Versuchungen zu widerstehen. |
| Adaptation | **[1]** | **Ich kann auf Veränderungen flexibel reagieren.** |
|  | **[2]** | **Auf Veränderungen (z.B. eine Planänderung) kann ich mich problemlos einstellen.** |
|  | **[3]** | **Es fällt mir schwer, mich spontan an Veränderungen anzupassen.*** |
| Abwägen | **[1]** | **Ich durchdenke sorgfältig alle Möglichkeiten, bevor ich etwas tue.** |
|  | **[2]** | **Ich handle, ohne alle Alternativen durchdacht zu haben.*** |
|  | **[3]** | **Ich handele, ohne nachzudenken und mache das Erste, das mir in den Sinn kommt.*** |

Nites: Bold items – final item set, ^*^ inversed items

Table S_2. Correlations between the shortened subscales of the Executive Functions in Healthy Adults Questionnaire (15 items), conscientiousness and moderate-to-vigorous physical activity (MVPA) in Study 2.

|  | 1 | 2 | 3 | 4 | 5 |
| --- | --- | --- | --- | --- | --- |
| 1 - EF flexibility |  |  |  |  |  |
| 2 - EF focusing | .33 |  |  |  |  |
| 3 - EF inhibition | *.07* | .21 |  |  |  |
| 4 - EF adaptation | .25 | .29 | *.04* |  |  |
| 5 - EF weighing | *.02* | .19 | .32 | *-.07* |  |
| 6 - conscientiousness | .19 | .37 | .31 | *.05* | .36 |
| 7 – impulsivity urgency | .18 | .33 | .52 | .22 | .33 |
| 8 – impulsivity lack of premeditation | *.03* | .19 | .32 | -.12 | .69 |
| 9 – impulsivity lack of perseverance | .29 | .45 | .36 | .22 | .27 |
| 10 – impulsivity sensation seeking | -.18 | *-.08* | *.08* | -.30 | .12 |
| 11 – grit consistency of interests | .13 | .38 | .36 | .19 | .29 |
| 12 – grit perseverance of effort | .31 | .45 | .33 | .21 | .30 |

*Notes:* bold: p < .05; *italic*: p > .05.

Table S_3. Descriptive statistics of the Executive Functions in Healthy Adults Questionnaire subscales in Study 3 (*N* = 609).

| **EF subscale** | **M** | **SD** | **Min** | **Max** |
| --- | --- | --- | --- | --- |
| EF flexibility | 4.07 | .86 | 1.00 | 6.00 |
| EF focussing | 3.56 | .91 | 1.00 | 6.00 |
| EF inhibition | 3.94 | .90 | 1.00 | 6.00 |
| EF adaptation | 4.08 | .94 | 1.00 | 6.00 |
| EF weighing up | 4.26 | .90 | 1.33 | 6.00 |
